# *Campylobacter* Colonisation of Poultry Slaughtered at Nigerian Slaughterhouses: Prevalence, Antimicrobial Resistance, and Risk of Zoonotic Transmission

**DOI:** 10.3390/tropicalmed10090265

**Published:** 2025-09-17

**Authors:** Emmanuel O. Njoga, Philip P. Mshelbwala, Akwoba J. Ogugua, Excel C. Enemuo-Edo, Onyinye S. Onwumere-Idolor, Temitope M. Ogunniran, Sunday N. Bernard, Joel C. Ugwunwarua, Ebube C. Anidobe, Chinwe E. Okoli, Enid Godwin, Simon I. Enem, James W. Oguttu

**Affiliations:** 1 Department of Agriculture and Animal Health, College of Agriculture and Environmental Sciences, University of South Africa, Florida 1710, South Africa; joguttu@unisa.ac.za; 2 Department of Veterinary Public Health and Preventive Medicine, Faculty of Veterinary Medicine, University of Nigeria, Nsukka 410001, Enugu State, Nigeria; ogugua.akwoba@unn.edu.ng (A.J.O.); exceledo08@gmail.com (E.C.E.-E.); johelconsults@gmail.com (J.C.U.); anidobeebube@gmail.com (E.C.A.); 3 Faculty of Veterinary Medicine, University of Abuja, Abuja 900109, Nigeria; okoli.chinwe@uniabuja.edu.ng (C.E.O.); enid.godwin@uniabuja.edu.ng (E.G.);; 4 Department of Primary Industries, Orange 2800, Australia; 5 Department of Animal Production, Faculty of Agriculture, Southern Delta University, PMB 005, Ozoro 501103, Delta State, Nigeria; onwumere-idolors@dsust.edu.ng; 6 Department of Veterinary Medicine, Faculty of Veterinary Medicine, University of Nigeria, Nsukka 410001, Enugu State, Nigeria; temitope.ogunniran@unn.edu.ng; 7 Department of Animal Production and Health, Faculty of Agriculture, Federal University Oye-Ekiti, Oye-Ekiti 371023, Ekiti State, Nigeria; drsunny3030@gmail.com

**Keywords:** antimicrobial-resistant *Campylobacter*, foodborne zoonosis, multidrug-resistant *Campylobacter*, poultry, zoonotic *Campylobacter* epidemiology, *Campylobacter* transmission risks

## Abstract

Zoonotic *Campylobacter* species (ZCS), particularly *C. jejuni* and *C. coli*, cause major foodborne gastroenteritis and poultry is the principal reservoirs. However, there is limited data on *Campylobacter* transmission risk practices and antimicrobial resistance (AMR) in Nigeria. Therefore, this study determined the prevalence, AMR, and risk practices aiding *Campylobacter* transmission in two major slaughterhouses processing poultry carcasses in Enugu State, Nigeria. Four hundred poultry faecal samples were analysed for zoonotic *Campylobacter* organisms using standard protocols. Antimicrobial resistance was profiled via Kirby–Bauer disk diffusion technique, against eight antimicrobial agents. Risk practices were assessed through slaughterhouse observations and interviews with 56 workers. The overall prevalence of *Campylobacter* infections was 14.5% (58/400), while the species-specific prevalence were 13% (52/400) and 1.5% (6/400) for *C. coli* and *C. jejuni*, respectively. *Campylobacter* colonisation was significantly higher (*p* < 0.05) in broilers, and during the wet season. The AMR profile of the isolates against the eight antibiotics tested was: Amoxicillin/clauvlanic acid (100%), vancomycin (100%), tetracycline (96.6%), ciprofloxacin (55.2%), chloramphenicol (44.8%), ceftazidime (10.3%), azithromycin (3.4%) and streptomycin (3.4%). All the 58 *Campylobacter* isolates were multidrug-resistant. The multiple antibiotic resistance indices ranged from 0.4 to 0.9, with a mean of 0.7. Major risk practice associated with ZCS transmission include non-use of personal protective equipment (100%), slaughtering on unsanitary surfaces (100%), using visibly unclean water for meat processing (100%), improper manual evisceration (75%), eating or drinking during processing (64.4%), slaughtering sick animals (37.5%), inadequate cleaning of surfaces and equipment after use (21.4%) and consuming raw meat during carcass processing (19.6%). The findings reflect critical gaps in food safety, occupational health, prudent antimicrobial use in poultry farming and zoonotic disease control, emphasizing the need for antibiotic regulation, training on hygienic meat processing, public education, infrastructural development of slaughterhouse facilities, and inter-sectorial collaboration to curb *Campylobacter* contamination and spread of antimicrobial resistance.

## 1. Introduction

The genus *Campylobacter* comprises a group of Gram-negative, spiral-shaped, motile bacteria well known as major causes of bacteria foodborne zoonoses globally. Among the numerous species within this genus, *Campylobacter jejuni* and *C. coli* are of predominant public health significance due to their strong association with human gastroenteritis, enterocolitis and other life threatening post-infection sequellae, especially in immune-compromised (children, the elderly and pregnant women) and occupationally at-risk persons (veterinarians, animal attendants, livestock farm workers) [1,2]. These organisms are primarily transmitted through the consumption of contaminated edible animal-derived foods, especially undercooked poultry products [3,4]. Transmission of zoonotic pathogens, particularly *C. jejuni* and *C. coli*, can also occur through direct or indirect contact with infected animals and their environments, especially in occupationally exposed populations [5,6,7,8,9]. The World Health Organization (WHO) has classified *Campylobacter* as one of the four key global causes of diarrhoeal diseases, thereby emphasizing the need for continuous surveillance, particularly in some low- and middle-income countries (LMICs), where regulation of food safety and hygienic practices in food-animal production and processing value chains may be suboptimal [10,11,12].

Poultry is the principal reservoir of ZCS [3,13]. Colonization of the avian gastrointestinal tract occurs asymptomatically, with bacterial loads often exceeding 10^6^ CFU/g in the caeca [14]. As a result, the risk of carcass contamination during processing is high, especially in informal or poorly regulated slaughterhouses where biosecurity and hygiene standards are frequently compromised. In Nigeria, food-producing animal production and the carcass processing are important component of the agricultural subsector, both as a means of food security and sustenance of livelihood [15,16,17]. However, the informal nature of most poultry slaughter operations, including those at the Ipka and Artisan slaughterhouses in Enugu State, Nigeria, where the study were conducted, could facilitate the persistence and dissemination of ZCS along the meat processing value chain [12].

Numerous studies have reported a high prevalence of ZCS in poultry slaughtered for human consumption, retail poultry meat and live bird markets across Nigeria and other sub-Saharan African countries [18,19,20,21,22]. However, there remains a paucity of data concerning the specific risk practices within poultry slaughter environments that potentiate ZCS transmission to humans. Slaughterhouse workers constitute an occupationally exposed population, as they are frequently in contact with live birds, poultry faecal matters, gut contents, feathers, and carcasses. Activities such as evisceration, de-feathering, and carcass handling, when conducted without appropriate personal protective equipment (PPE) and under inadequate sanitary conditions, can result in direct transmission of zoonotic pathogens, including *Campylobacter*, to humans through contact with dermal and mucosal abrasions [23,24].

Beyond the direct public health implications of *Campylobacter* infections, there is a growing global concern regarding the antimicrobial resistance (AMR) conundrum associated with zoonotic pathogens, especially *Campylobacter* species. The emergence of multidrug-resistant pathogens, especially *Campylobacter* strains, has been increasingly documented, especially in LMICs [6,25,26], where antimicrobial use in animal agriculture is indiscriminate, rampant and poorly regulated [27,28,29]. Critically important antibiotics classified as “reserve” antimicrobials according to the WHO’s AWaRe classification [30], such as fluoroquinolones (e.g., ciprofloxacin) and macrolides (e.g., azithromycin), are commonly used at sub-therapeutic doses for growth promotion and prophylaxis in poultry production in Nigeria [27,28], despite being frontline drugs for treatments for severe campylobacteriosis. This practice creates a selective pressure that facilitates the proliferation of antimicrobial-resistant strains of bacteria, thereby complicating therapeutic outcomes in animal and human microbial infections and contributing to the broader global AMR crisis [31,32,33].

Understanding the resistance patterns of zoonotic *Campylobacter* isolates from poultry sources is, therefore, critical for the formulation of effective and tailored antimicrobial stewardship programs, and for guiding empirical treatment decisions in clinical settings. Moreover, determining prevalence of ZCS in poultry slaughtered for human consumption and the slaughterhouse practices that could aid the zoonotic spread of the pathogens provide evidence base information for targeted interventions aimed at breaking the transmission cycle. The Ikpa and Artisan slaughterhouses, serving a substantial proportion of the poultry-consuming population in Southeast Nigeria, represents a pertinent case study for assessing the risks of ZCS contamination and infection associated with poultry slaughter and the occupational hazards faced by slaughterhouse workers in their workplace.

Therefore, the objectives of this study are to (1) ascertain the prevalence (faecal carriage) of *C. jejuni* and *C. coli* in poultry processed for human consumption, (2) characterise the phenotypic AMR profiles of the isolates, and (3) evaluate slaughterhouse practices that could enhance zoonotic transmission of *Campylobacter* species in slaughterhouse setting in Nigeria. Given Nigeria’s public health landscape, characterised by underreported foodborne illnesses, studies like this are essential for developing targeted interventions to support national compliance with international food safety and AMR guidelines, such as those outlined by the WHO Global Action Plan and the FAO [34,35]. Findings from this study could guide policy-making to enhance the microbiological safety of poultry meat in Nigeria, thereby reducing the risk of exporting antibiotic-resistant *Campylobacter* pathogens globally.

## 2. Materials and Methods

### 2.1. Ethical Approval and Informed Consent

The Institutional Animal Care and Use Committee at the Faculty of Veterinary Medicine, University of Nigeria, Nsukka, granted ethical approval for this research, referenced under FVM-UNN-IACUC-2025-03/215. Informed oral consent was solicited and secured from all 56 slaughterhouse workers who voluntarily participated in the interview process. The participants were all individuals over the age of 18 years. No minors were involved in the study.

### 2.2. Study Area and Design

This research was carried out at Ikpa and Artisan slaughterhouses, which are the major municipal facilities for poultry slaughter and processing in Enugu State, Nigeria. The demographic characteristics, geographical setting, and orographic features of Enugu State, where these slaughterhouses are situated, have been previously documented [36,37,38]. A cross-sectional study design was employed, focusing on the isolation of *Campylobacter* species from poultry faecal samples, the antimicrobial susceptibility profiling of phenotypically identified *C. jejuni and C. coli*, as well as an evaluation of poultry carcass processing practices among the slaughterhouse workers.

### 2.3. Sample Size Determination and Sample Collection

Using the Raosoft^®^ sample size calculator available at http://www.raosoft.com/samplesize.html (accessed on 21 March 2023) [39], a minimum sample size of 236 poultry faecal samples was computed for this study. This calculation was based on a prevalence of 18.9%, as reported by Njoga et al. [7], a 5% margin of error and an estimated poultry population exceeding 20,000 in the area of study. Two slaughter points were selected in each slaughterhouse by simple random sampling (toss of coin). At each selected slaughter point, systematic random sampling (with 1 in 4) was used to select poultry carcasses to be sampled. Consequently, a total of 400 faecal samples were randomly collected) and investigated for the presence of *C. jejuni* and *C. coli*. Using sterile swabs moistened in aseptically prepared nutrient broth (CMO001B, Oxoid, UK) according to the manufacturer’s instruction, the faecal samples were collected directly from the caeca post-evisceration and stored in sample containers filled with ice packs. Afterwards, the stool samples were conveyed, in cold condition, to the laboratory for *Campylobacter* isolation within three hours of sample collection. The sample collection lasted between January and August 2024.

### 2.4. Isolation of Zoonotic Campylobacter Species

The isolation of *Campylobacter* species was conducted utilizing Modified Charcoal Cefoperazone Deoxycholate Agar (mCCDA, CMO739, Oxoid, UK), following the methodology outlined by Hagos et al. [40], albeit with minor modifications. The mCCDA medium was prepared in accordance with the manufacturer’s guidelines and augmented with a specific *Campylobacter* supplement (SRO 155E, Oxoid, UK) to enhance bacterial isolation. Swab samples were streaked directly onto the supplemented agar, which was then incubated under microaerophilic conditions (5% O2, 10% CO2, and 85% N2) achieved through the use of CampyGen^®^ (CN 0025A, Oxoid, UK) at a temperature of 42 °C for a duration of 48 h.

Plates that showed microbial growth were evaluated for presence of suspected *Campylobacter* colonies characterized by flattened, thinly spreading, and creamy morphology (indicative of *C. jejuni*) or glossy appearance with pinpoint size (indicative of *C. coli*) on the agar surface. The suspected *Campylobacter* colonies were subsequently purified on supplemented mCCDA plates under the same incubation conditions. Following purification, putative pure colonies underwent biochemical characterization in accordance with the protocols established by Barrett et al. [41]. Colonies that yielded positive results in biochemical assays were subjected to Gram staining and microscopically examined using the oil-immersion objective. The identification of slender, short Gram-negative rods exhibiting curved, spiral, comma, or S-shaped morphology were assumed to be putative *Campylobacter* species.

### 2.5. Antimicrobial Resistance Profiling

#### 2.5.1. Antibiogram

The antimicrobial susceptibility of the *Campylobacter* species was assessed using the Kirby–Bauer agar disc diffusion method as described by Shakir et al. [42]. The sensitivity profiles of the isolates were evaluated against eight commonly employed antimicrobial agents spanning eight classes of antibiotics. The specific antimicrobial discs used, along with their respective concentrations, included tetracycline (30 µg), amoxicillin/clavulanic acid (30 µg), vancomycin (30 µg), azithromycin (15 µg), streptomycin (10 µg), ceftazidime (30 µg), chloramphenicol (30 µg), and ciprofloxacin (5 µg), all sourced from Oxoid, UK. A suspension of each test isolate was prepared at a 0.5 McFarland turbidity standard and evenly distributed on Mueller-Hinton agar (CM0337, Oxoid, UK) plates. Antimicrobial discs were then gently placed, and well-spaced out, on the surface of the agar, and the setup was allowed to rest at ambient temperature for 30 min to facilitate antibiotic diffusion prior to incubation under microaerophilic conditions at 37 °C for 24 h.

The diameters of the inhibition zones surrounding each disk were measured in millimetres and interpreted in accordance with the criteria established by the Clinical and Laboratory Standards Institute (CLSI) [43]. Based on the measured inhibition zone diameters, the AMR profiles were classified as susceptible (S), intermediate (I), or resistant (R). However, intermediate inhibition zones were reported as resistant, in light of CLSI recommendations to adopt a more conservative interpretation of resistance status, particularly due to the risk of clinical treatment failure associated with intermediate-resistant organisms in standard dosing regimens, especially within immunocompromised populations or in infections located in anatomical sites where drug penetration may be suboptimal [44,45].

#### 2.5.2. Multiple Antimicrobial Resistance Indices

The Multiple Antimicrobial Resistance (MAR) index, defined as the ratio of the number of antibiotics to which an isolate exhibits resistance relative to the total number of antibiotics tested, was computed using the formula: MAR Index = R/E, as described by Shakir et al. [42]. In this formula, R denotes the total number of antibiotics that the organism is resistant to, while E represents the total number of antibiotics to which the organism was exposed. An MAR index exceeding 0.2 is indicative of a high-risk source of contamination (Shakir et al. [42]). Furthermore, the multidrug resistance status, described as non-susceptible to at least one antimicrobial agent in three or more classes of antimicrobials that are structurally and mechanistically different, was also determined based on the antimicrobial susceptibility patterns of the isolates [46].

### 2.6. Risk Practices

A comprehensive study was conducted to identify the risk practices and behavioural patterns of slaughterhouse workers that may contribute to the zoonotic transmission of *Campylobacter* species. This was achieved through direct observation of the workers during their routine carcass processing operations. The findings, which revealed noteworthy insights into their practices, were captured on camera and documented through vivid imagery. To quantify the proportion of the slaughterhouse workers that took part in certain risk practice aiding ZCS transmission, a census of all the workers that reported to duty on each research visit day was determined. Workers who consented to partake in the study were interviewed, to determine the practices they were involved in. The interview was done in the local dialect (Igbo) for workers who were not proficient in their understanding of the English Language.

### 2.7. Statistical Analyses

Fisher’s exact test was utilized to assess the statistical association (*p* < 0.05) between *Campylobacter* colonization, as the outcome, and various epidemiological variables as explanatory variables, which included poultry types, seasonal factors, and slaughterhouse locations. This statistical method was similarly employed to evaluate the antimicrobial susceptibility profiles of *C. jejuni* and *C. coli* against the eight antimicrobial agents under investigation. All analyses were conducted at a significance level of 5% using GraphPad Prism^®^ version 6.04 (GraphPad^®^ Inc., San Diego, CA, USA).

## 3. Results

### 3.1. Prevalence of Campylobacter Species

*Campylobacter* colonisation was significantly higher (*p* < 0.05) in broilers, with a prevalence of 17.9% (50/280), compared to other poultry types, which had a prevalence of 6.7% (8/120). Similarly, *Campylobacter* colonisation was significantly (*p* < 0.05) higher during the rainy/wet season. The detailed distribution of ZCS isolated according to epidemiological variables of interest is summarized in Table 1.

### 3.2. Results on Antimicrobial Resistance

#### 3.2.1. Antibiogram

**Figure 1:**
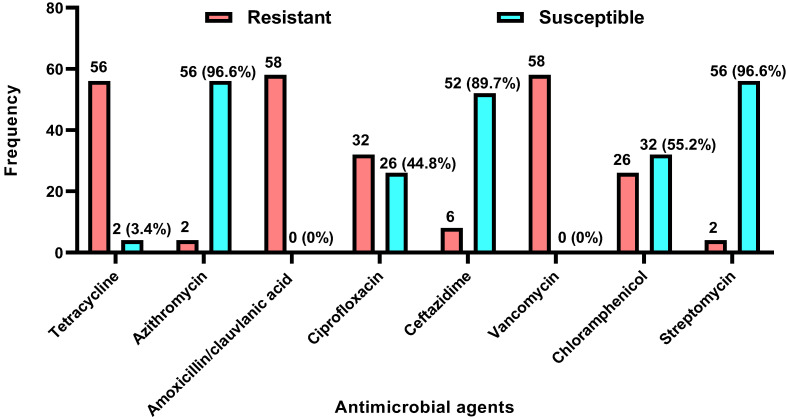
Overall distribution of the antimicrobial resistance profile of *Campylobacter* species (n = 58) isolated from poultry slaughtered for human consumption at municipal slaughterhouses in Enugu State, Nigeria.

All 58 *Campylobacter* isolates demonstrated multidrug resistance, showing non-susceptibility to at least one antimicrobial agent in three or more distinct antimicrobial classes with different structural and mechanistic properties. All isolates were non-susceptible to both amoxicillin/clavulanic acid (30 µg) and vancomycin (30 µg). The most effective antimicrobial agents against the isolates were azithromycin (96.6%, 56/58), streptomycin (96.6%, 56/58), ceftazidime (89.7%, 52/58), chloramphenicol (55.2%, 32/58), and ciprofloxacin (44.8%, 26/58), as shown in Figure 1.

Comparative analysis of AMR profiles reveals important differences. All *C. jejuni* isolates were resistant to ciprofloxacin, a first-line therapeutic agent for clinical management of complicated human campylobacteriosis, and tetracycline. However, all *C. jejuni* isolate were susceptibility to ceftazidime (Table 2). In stark contrast, a substantial proportion of *C. coli* isolates exhibited resistance, with 50% demonstrating non-susceptibility to ciprofloxacin and a striking 96.2% showing resistance to tetracycline, as detailed in Table 2.

#### 3.2.2. Multiple Antimicrobial Resistance Indices and Antimicrobial Resistance Patterns

**Figure 2:**
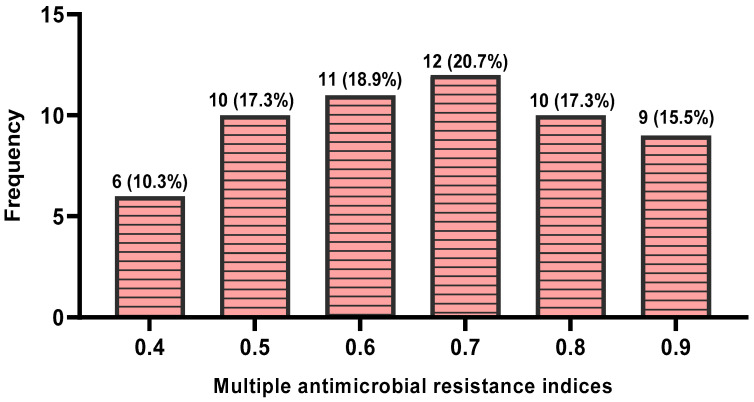
Distributions of various MAR indices of *Campylobacter* species isolated from poultry slaughtered at slaughterhouses in Enugu State, Nigeria.

The isolates exhibited MAR indices values which ranged from 0.4 to 0.9, with an average MAR index of 0.7. Detailed results on the distributions of various MAR indices of the isolates are shown in Figure 2.

The 58 *Campylobacter* isolates exhibited 11 distinct AMR phenotypic resistance patterns (Table 3). The most prevalent was the AMC-TET-VAN pattern, found in 10 isolates (17.2%). The most extensive was the AMC-CRL-CIP-TET-STR-VAN pattern, observed in two isolates.

#### 3.2.3. Practices that Heighten the Risk of Zoonotic *Campylobacter* Transmission

Direct observation during carcass processing and interviews with 56 slaughterhouse workers documented widespread risky practices. None of the workers (100%, 56/56) wore PPE. All slaughtering was performed on bare ground or unsanitary floors (100%, 56/56), and visibly unclean or non-potable water was used for processing (75%, 42/56). Improper manual evisceration (75%, 42/56) and eating, smoking, or drinking during processing (64.4%, 36/56) were also common risk practices.

**Figure 3:**
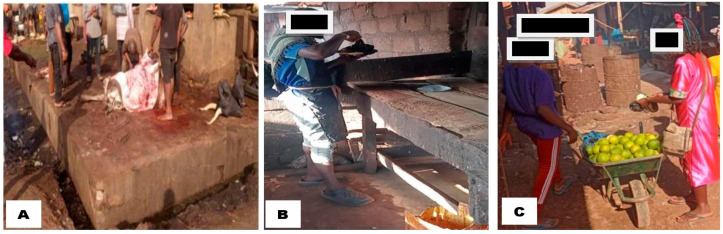
Images showing where slaughterhouse workers are seen standing on barefoot while processing carcass (**A**). One worker was observed eating within the facility (**B**), while another patiently waited to collect oranges purchased from a vendor at the slaughterhouse (**C**).

Other *Campylobacter* transmission and infection risk practices included consumption of raw meat (19.6%, 11/56), slaughtering clinically sick animals (37.5%, 21/56), and inadequate cleaning of surfaces and equipment after use (21.4%, 12/56). Pictorially documented risk practices were shown in Figure 3.

## 4. Discussion

### 4.1. Prevalence of Zoonotic Campylobacter Species

The isolation of ZCS in 14.5% of poultry slaughtered for human consumption is a significant finding from a One Health perspective because of the potential of the carcasses acting as a source of human *Campylobacter* infection or contaminating the environment. The zoonotic risk posed by *C. jejuni* and *C. coli*, coupled with their ease of transfer through the food supply or through occupational exposure, highlights from a public health and food safety point of view, the importance of the observed 14.5% overall prevalence. These results suggest an elevated risk of human infection with ZCS, particularly in poultry farming communities where humans and animals often cohabit. This practice of animal–human cohabitation is common among smallholder poultry farmers in resource-limited settings and regions [47], including Nigeria. Furthermore, many slaughterhouse workers in Nigeria typically do not use personal protective equipment, making them vulnerable to infections from zoonotic pathogens, such as ZCS, during the processing of carcasses [12,38,48].

The *C. jejuni* and *C. coli* isolated in this study are implicated in 95% of human campylobacteriosis cases and are the principal cause of foodborne gastroenteritis in people [21,49,50]. Although the mortality rates associated with human campylobacteriosis is generally low among healthy adults [51], ZCS, particularly *C. jejuni*, can cause severe post-infection complications, including Guillain-Barré syndrome, Miller Fisher syndrome, reactive arthritis, Reiter’s syndrome, irritable bowel syndrome, inflammatory bowel disease, Crohn’s disease, and ulcerative colitis [52].

From a food safety perspective, the 14.5% prevalence is epidemiologically significant, particularly considering the role of poultry as the primary reservoir of ZCS, and the increased risk of carcass contamination during processing. Nevertheless, this risk is partially mitigated by the widespread cultural practice in many African communities of thoroughly cooking meat, which substantially reduces bacterial load and minimizes transmission potential. Typically, meat is boiled, fried, or grilled at temperatures between 80 and 90 °C for up to 20 min or longer [53]. Such cooking methods effectively eradicate most bacterial contaminants, thus significantly reducing the chances of *Campylobacter* transmission through the food supply and helping to prevent foodborne illness outbreaks in numerous African communities.

While poultry serves primarily as a reservoir for ZCS responsible for human campylobacteriosis, a newly identified species, *C. hepaticus*, causes spotty liver disease in poultry, resulting in up to 15% mortality rates and a 35% decline in egg production in layers [54]. Additionally, since poultry are frequently raised in proximity to other domestic animals such as small ruminants and pets, especially among smallholder farmers in rural areas, there exists a possibility of *Campylobacter* transmission between animals or from animals to humans. Similarly to their impact on humans, *Campylobacter* species can cause health problems in animals and hence economic loses. More importantly, *C. jejuni* can cause late-term abortions in small ruminants, particularly in sheep, leading to significant economic consequences and potential zoonotic transmission pathways for the pathogen [55,56,57].

The overall 14.5% prevalence of ZCS reported is lower than the 36% documented by Akwuoba et al. [58] in the same geographical area in 2010. The reduction in prevalence may be attributed to the widespread implementation of intensive poultry farming practices, which limit exposure of birds to high environmental temperatures that typically encourage the spread of infections associated with ZCS, which are known to thrive in warmer conditions [4,59,60,61]. Moreover, these farming systems often prevent the intrusion of wild or migratory birds, which are natural reservoirs of ZCS for infection of domestic poultry [7]. Additionally, the extensive use of prophylactic antimicrobials in poultry farming throughout the country [27,28] may have contributed to the observed lower prevalence of *Campylobacter* infections. While targeted therapeutics usually prevent clinical illness in poultry, the use of antimicrobial agents for the prophylactic management of other economically significant bacterial diseases may inadvertently suppress levels of non-target pathogens like *Campylobacter*. As a result, this could lead to reduced colonization of *Campylobacter* in poultry, which helps explain the low prevalence observed.

At the global level, the 14.5% prevalence is comparatively lower than the 36.2% in Peru [62], 43.1% in Ghana [63], 79.2% in the UK [64], 32.5% in Romania [65], 70% in Ethiopia [66], and 25.1% in Iran [67]. These variations may be attributed to differences in diagnostic methodologies, researcher expertise, farm-level biosecurity and hygiene practices, as well as underlying epidemiological factors influencing *Campylobacter* transmission dynamics across the various study locations.

### 4.2. Antimicrobial Resistance

With all isolates displaying multidrug resistance, the AMR status of ZCS from poultry in the study area is very high. This level of resistance, particularly against amoxicillin/clavulanic acid, tetracycline and vancomycin, carries significant public health implications. The 11 AMR patterns identified among the 58 ZCS isolates highlight the pronounced antimicrobial-resistant capabilities of these pathogens. The high prevalence of AMR is a considerable public health issue [59,68,69], especially since beta-lactams and tetracycline are commonly used to treat suspected *Campylobacter*-induced gastroenteritis in Nigeria and other developing countries, despite their reduced efficacy compared to macrolides and fluoroquinolones, which are the drugs of choice for clinical treatment of campylobacteriosis [6].

The MAR indices, which ranged from 0.4 to 0.9, with an average of 0.7, signify a substantial threat to public health. A MAR index exceeding 0.2 suggests exposure to high-risk environments where antibiotic usage is excessive and often inappropriate [70,71]. Hence, the high mean value of 0.7 indicates widespread resistance across various antibiotic classes, potentially undermining the effectiveness of commonly prescribed antibiotics for *Campylobacter*-related infections in humans, including macrolides and fluoroquinolones. This limitation could significantly reduce treatment options and heighten the risks of treatment failure, prolonged illness, and complications, especially for vulnerable populations. This situation underscores the urgent need for enhanced antibiotic stewardship in poultry farming, routine surveillance of antimicrobial resistance, and the implementation of One Health-based strategies to control the dissemination of antibiotic-resistant ZCS from food-producing animals, particularly poultry.

The high AMR prevalence of 100% noted against amoxicillin/clavulanic acid is consistent with the findings from Okunlade et al. [72] in Oyo State, Nigeria, where all *Campylobacter* isolates from poultry exhibited resistance to amoxicillin, ceftriaxone, and erythromycin. The AMR prevalence of 55.2% to ciprofloxacin among the *Campylobacter* isolates is clinically significant, given that this antimicrobial agent is preferred for treatment for campylobacteriosis [73]. The elevated resistance levels may be attributed to the overuse and misuse of antibiotics in Nigeria, where ciprofloxacin, classified as “Reserve group” antimicrobials in the WHO’s “AWaRe” categorization [30], are often available over the counter and sometimes used for chemoprophylaxis [27,28]. Factors contributing to AMR development in zoonotic bacteria like *Campylobacter* in developing countries include improper prescription practices, insufficient patient education, limited diagnostic capabilities, unauthorized sales of antimicrobials, and weak regulatory frameworks [74,75]. Furthermore, the rise in globalization and international travel enhances the risk of transmitting these antibiotic-resistant pathogens [6]. Therefore, addressing AMR through a One Health approach in Africa and other developing regions is vital to prevent the global spread of antimicrobial-resistant pathogens [6], especially *Campylobacter*.

In addition to adverse health outcomes, antimicrobial-resistant *Campylobacter* species could lead to significant economic loses, as a result of prolonged periods of hospitalizations and prescription of last-resort drugs, which are often very costly [76,77]. Severe human infections can even result in mortality or long-term health issues [6], while livestock can suffer substantial production losses, including late-term abortions, stillbirths, and various infertility challenges [56,57].

### 4.3. Zoonotic Campylobacter Transmission Risk Factors

The engagement in risky practices that have potential to enhance transmission of zoonotic *Campylobacter* among poultry processors in Nigeria has grave One Health implications. The observed high prevalence of risk behaviours such as non-use of personal protective equipment (100%), slaughtering on bare or unsanitary floors (100%), and use of visibly unclean water for carcass processing (100%) highlights the high potential for exposure of processors and consumers to *Campylobacter* spp., notably *C. jejuni* and *C. coli*. These pathogens are among the most common bacterial causes of gastroenteritis globally, often transmitted through direct contact with contaminated animal products or surfaces [1,78]. The absence of PPE facilitates direct skin contact with contaminated blood, faeces, and viscera, while unsanitary slaughtering surfaces and water create foci for pathogen persistence and transmission. Such environmental contamination not only increases the risk of enteric infections in humans but also contributes to the spread of pathogen through flies, rodents, and runoff water, thus reinforcing environmental health risks [79,80].

The observed poor manual evisceration technique adopted by majority of workers (75%) and unhygienic habits like eating, drinking, or smoking during carcass processing compound the risk of faecal-oral transmission. Poorly performed evisceration increases the likelihood of intestinal rupture, leading to faecal contamination of meat with *Campylobacter*, known for its low infectious dose (as few as 500 organisms) [81]. The consumption of undercooked or raw meat (19.6%) is an additional direct route for infection, while the slaughtering of clinically sick animals (37.5%) introduces pathogens into the food chain, many of which may harbour antimicrobial resistance traits [2]. From a One Health perspective, the poor sanitation of equipment and facilities (21.4%) creates a sustained cycle of infection and recontamination, impacting not only human and animal health but also contaminating shared environmental resources like soil and water bodies [82]. These cumulative practices reflect a critical gap in food safety, occupational health, and zoonotic disease control, emphasizing the need for integrated interventions through a One Health framework that includes training, regulation, and infrastructure development.

## 5. Conclusions and Recommendations

The detection of zoonotic *Campylobacter* species (*C. jejuni* and *C. coli*) in 14.5% of poultry slaughtered for human consumption at two major municipal abattoirs in Enugu State, Nigeria, presents a substantial concern from One Health perspectives. The fact that all the isolates exhibited multidrug resistance underscores the urgent need for judicious antimicrobial use within animal agriculture, particularly in the poultry sector, to curb the emergence and dissemination of antimicrobial-resistant zoonotic *Campylobacter* strains. Promoting enhanced on-farm hygiene and the implementation of stringent biosecurity measures could reduce reliance on antibiotics in poultry production. Notably, the adoption of gut microbiota modulators, such as gut balance boosters, which are proven alternatives to antibiotic growth promoters in livestock production [83] offers a promising strategy. Moreover, the prevalent risky practices associated with zoonotic *Campylobacter* transmission observed among poultry processors, reflects a critical gap in food safety, occupational health, and zoonotic disease prevention systems within the region. This highlights the pressing need for comprehensive, integrated interventions grounded in a One Health approach. Such strategies should encompass capacity building through targeted training, the establishment and enforcement of regulatory frameworks, and the development of essential infrastructure to support safe and sustainable poultry production and processing.

## 6. Limitation of the Study

Molecular technique, such as PCR, was not employed in the diagnosis of phenotypically identified *Campylobacter* species. Nevertheless, the findings remain robust and provide valuable insights that contribute meaningfully to global One Health improvement, particularly in LMICs.

## Figures and Tables

**Table 1:** Distribution of zoonotic *Campylobacter* species from poultry faecal samples collected at Nigerian slaughterhouses according to various epidemiological variables.

Epidemiological Variables	Levels	Number Tested	Number Positive (*Campylobacter* Isolated)	Number Negative (*Campylobacter* Not Isolated)	*p*-Value
Poultry type	Broiler	280	50	232	0.0031 *
	Others †	120	8	112	
Season	Rainy/wet	220	46	174	0.0001 *
	Dry/hot	180	12	168	
Slaughterhouse location	Nsukka	208	31	177	0.7784
	Enugu	192	27	165	

† = layers, cockerel, turkey and indigenous chicken; * = statistically significant *p*-value; Fisher’s exact test (GraphPad Prism^®^, version 8.0.4, San Diego, CA, USA).

**Table 2:** Comparative frequency distribution of the antimicrobial resistance status of *C. jejuni* and *C. coli* isolated from poultry slaughtered for human consumption at municipal slaughterhouses in Enugu State, Nigeria.

Antimicrobial Agent	*Campylobacter jejuni* (n = 6)		*Campylobacter coli* (n = 52)		*p*-Value
	Resistant (%)	Susceptible (%)	Resistant (%)	Susceptible (%)	
Tetracycline (30 µg)	6 (100)	0 (0)	50 (96.2)	2 (3.8)	0.0999
Azithromycin (15 µg)	0 (0)	6 (100)	2 (3.8)	50 (96.2)	0.0999
Amoxicillin/Clavulanic acid (30 µg)	6 (100)	0 (0)	52 (100)	0 (0)	0.0999
Ciprofloxacin (5 µg)	6 (100)	0 (0)	26 (50)	26 (50)	0.0281 *
Ceftazidime (30 µg)	0 (0)	6 (100)	6 (11.5)	46 (88.5)	0.0999
Vancomycin (30 µg)	6 (100)	0 (0)	52 (100)	0 (0)	0.0999
Chloramphenicol (30 µg)	2 (33.3)	4 (66.7)	24 (46.2)	28 (53.8)	0.6814
Streptomycin (10 µg)	0 (0)	6 (100)	2 (3.8)	50 (96.2)	0.0999

* = Statistically significant *p*-value; Fisher’s exact test (GraphPad Prism^®^, version 8.0.4, San Diego, CA, USA).

**Table 3:** Antimicrobial resistance patterns of *Campylobacter* species isolates from poultry slaughtered for human consumption at municipal slaughterhouses in Enugu State, Nigeria.

S/n	Phenotypic Antimicrobial Resistance Pattern Exhibited	Frequency (%)
1.	AMC-CIP	2 (3.4)
2.	AZM-STR	2 (3.4)
3.	TET-CIP	5 (8.6)
4.	TET-CLR	5 (8.6)
5.	AMC-CIP-VAN	6 (10.3)
6.	CIP-CRL-VAN	8 (13.8)
7.	AMC-TET-VAN	10 (17.2)
8.	CAZ-CRL-CIP	6 (10.3)
9.	AMC-CIP-TET-VAN	6 (10.3)
10.	AMC-CRL-CIP-TET-VAN	6 (10.3)
11.	AMC-CRL-CIP-TET-STR-VAN	2 (3.4)

AMC = Amoxicillin/clavulanic acid, CAZ = ceftriaxone, CRL = chloramphenicol, CIP = ciprofloxacin, VAN = vancomycin, AZM = azithromycin, STR = streptomycin, TET = tetracycline.

## Data Availability

All data generated in this study is with the published paper.
